# Assessing signs of central sensitization: A critical review of physiological measures in experimentally induced secondary hyperalgesia

**DOI:** 10.1002/ejp.4733

**Published:** 2024-09-24

**Authors:** Caterina M. Leone, Cedric Lenoir, Emanuel N. van den Broeke

**Affiliations:** ^1^ Department of Human Neuroscience Sapienza University of Rome Rome Italy; ^2^ Institute of Neuroscience, UCLouvain Brussels Belgium; ^3^ Department of Health Psychology KU Leuven Leuven Belgium

## Abstract

**Background and Objectives:**

Central sensitization (CS) is believed to play a role in many chronic pain conditions. Direct non‐invasive recording from single nociceptive neurons is not feasible in humans, complicating CS establishment. This review discusses how secondary hyperalgesia (SHA), considered a manifestation of CS, affects physiological measures in healthy individuals and if these measures could indicate CS. It addresses controversies about heat sensitivity changes, the role of tactile afferents in mechanical hypersensitivity and detecting SHA through electrical stimuli. Additionally, it reviews the potential of neurophysiological measures to indicate CS presence.

**Databases and Data Treatment:**

Four databases, PubMed, ScienceDirect, Scopus and Cochrane Library, were searched using terms linked to ‘hyperalgesia’. The search was limited to research articles in English conducted in humans until 2023.

**Results:**

Evidence for heat hyperalgesia in the SHA area is sparse and seems to depend on the experimental method used. Minimal or no involvement of tactile afferents in SHA was found. At the spinal level, the threshold of the nociceptive withdrawal reflex (RIII) is consistently reduced during experimentally induced SHA. The RIII area and the spinal somatosensory potential (N13‐SEP) amplitude are modulated only with long‐lasting nociceptive input. At the brain level, pinprick‐evoked potentials within the SHA area are increased.

**Conclusions:**

Mechanical pinprick hyperalgesia is the most reliable behavioural readout for SHA, while the RIII threshold is the most sensitive neurophysiological readout. Due to scarce data on reliability, sensitivity and specificity, none of the revised neurophysiological methods is currently suitable for CS identification at the individual level.

**Significance:**

Gathering evidence for CS in humans is a crucial research focus, especially with the increasing interest in concepts such as ‘central sensitization‐like pain’ or ‘nociplastic pain’. This review clarifies which readouts, among the different behavioural and neurophysiological proxies tested in experimental settings, can be used to infer the presence of CS in humans.

## 1 INTRODUCTION

Central sensitization (CS) is defined by the International Association for the Study of Pain (IASP) as the ‘*increased responsiveness of nociceptive neurons in the central nervous system to their normal or subthreshold input’*. CS is believed to contribute to many chronic pain conditions, suggesting a need for objective criteria for establishing CS (Arendt‐Nielsen et al., 2018; Nijs et al., 2021; Woolf, 2011). Following the IASP definition, CS can be measured by recording the responsiveness of nociceptive neurons in the central nervous system (CNS). The IASP defines a nociceptive neuron as ‘*a central or peripheral neuron of the somatosensory nervous system that is capable of encoding noxious stimuli’*. In humans, it is not possible, non‐invasively, to record from single nociceptive neurons in the CNS. The neurophysiological techniques that are available to record CNS activity in humans, including evoked potentials and reflexes, only record the activity of (large) pools of neurons and are probably unlikely to capture the activity of nociceptive specific neurons only.

Secondary hyperalgesia (SHA), the increase in evoked pain in the skin surrounding a cutaneous injury (Sandkühler, 2009), is the best‐documented manifestation of CS and can be induced experimentally. There are a dozen experimental methods to induce SHA, the description of which goes beyond the scope of this review and has been detailed elsewhere (Quesada et al., 2021).

The aim of this review was to critically discuss how the presence of CS evaluated by the manifestation of SHA affects physiological measures in healthy volunteers and ultimately if those measures could be used to infer the presence of CS.

We discussed the changes in perception elicited by stimuli belonging to different modalities, after the application of experimental methods used to induce SHA, with the aim to address three controversial issues: (1) Is there a change in heat sensitivity in the area of SHA? (2) Is there a contribution of tactile afferents to SHA? (3) Can we detect SHA using electrical stimuli?

The literature suggests mixed results regarding secondary heat hyperalgesia. An animal study on inflammatory arthritis tested whether SHA depends on the class of peripheral nociceptor (C‐ or A‐nociceptor) rather than the type of stimulation (mechanical vs. heat) (Hsieh et al., 2015). They found SHA was evoked by A‐nociceptor thermal stimulation, suggesting it is A‐nociceptor dependent, not stimulus modality dependent. We systematically searched the literature to investigate the consistency of secondary heat hyperalgesia.

Since mechanical pinprick stimuli activate low‐threshold mechanoreceptors (Cohen & Vierck, 1993; Johansson & Vallbo, 1979; Vallbo & Johansson, 1984), we explored whether these receptors contribute to changes in mechanical pinprick sensitivity.

Finally, previous studies suggest that secondary mechanical hyperalgesia is mediated by A‐fibre nociceptors (Ziegler et al., 1999). We explored whether electrical stimuli, supposed to selectively activate A‐fibre nociceptors, could identify secondary mechanical hyperalgesia.

Lastly, we reviewed the changes in different electrophysiological measures originating from the central and peripheral nervous systems, assessed in the context of experimentally induced SHA, to determine if those measures could be used to infer the presence of CS.

## 2 METHODS

**FIGURE 1:**
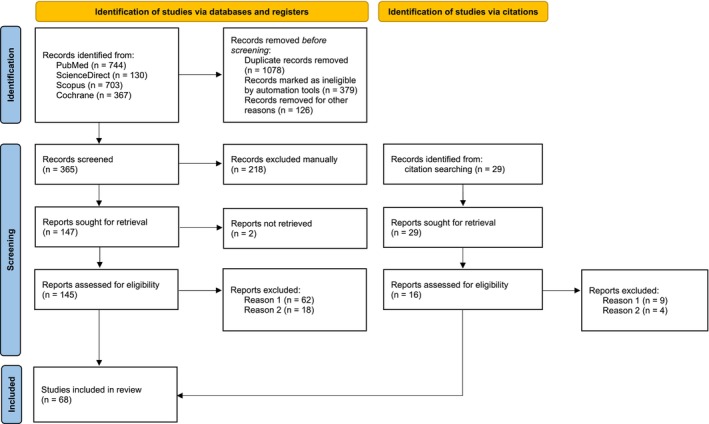
Flow diagram of the systematic searches of databases according to PRISMA guidelines (Page et al., 2021). Reason 1 refers to the type of article included (original research papers conducted in healthy volunteers) and reason 2 refers to the fact that the outcomes were obtained from stimuli delivered in the area of secondary hyperalgesia, which was identified using pinprick stimuli.

After an initial exploratory search according to the three different questions we aimed to address, and according to the changes in neurophysiological measures occurring in the context of SHA, we performed systematic research of the literature. We adopted a systematic approach to building this narrative review (see the flow diagram of systematic searches in Figure 1). Four databases including PubMed, ScienceDirect, Scopus and Cochrane Library were searched using the terms: ‘hyperalgesia’ or ‘secondary hyperalgesia’ or ‘pinprick hyperalgesia’ or ‘mechanical hyperalgesia’ and ‘human’. Search was limited to research articles conducted on healthy volunteers and published in English until 2023. Additionally, to specifically look for research articles focusing on our research questions and reporting on the neurophysiological outcomes we aimed to discuss, successive searches were then performed including the same research terms in addition to ‘thermal’ or ‘heat’ or ‘reflex’ or ‘tactile’ or ‘touch’ or ‘electrical’ or ‘potentials’ or ‘sympathetic’ or ‘pupil’ or ‘skin conductance’. After merging all records, we discarded duplicates. Reports were excluded if they were not original research articles (reason 1 in the flow diagram) and if the outcomes were not obtained by stimuli delivered in the area of secondary hyperalgesia delineated using pinprick stimuli (reason 2). Relevant reports cited in the screened research articles have been reviewed and considered eligible if matching the research criteria mentioned above. Each article was reviewed by at least two authors. If there was no agreement on the extracted results, a review by the third author was necessary. After this process, we finally included 68 studies in total.

## 3 BEHAVIOURAL CHARACTERISTICS OF SECONDARY HYPERALGESIA

SHA seems most prominent for sharp mechanical stimuli, such as pinprick stimuli (Meyer & Campbell, 1981). Given the fact that mechanical pinprick stimuli are often not perceived as painful in normal conditions, that is, when delivered to non‐sensitized skin, strictly speaking, the increase in perceived pinprick sensitivity after sensitization cannot be labelled as secondary hyperalgesia or allodynia (pain evoked by non‐nociceptive stimuli), and hyperesthesia would be preferable (IASP). Indeed, to label the increased perception of sharp mechanical punctate stimuli as hyperalgesia, those pinprick stimuli should be perceived as painful at baseline, that is, before sensitization is induced. To label the change in perception elicited by mechanical pinprick stimuli as allodynia those sharp indenting pinprick stimuli should not activate peripheral nociceptors, which is clearly the case (Greenspan & McGillis, 1991; Magerl et al., 1998; Ziegler et al., 1999) (see also dynamic allodynia related to the activation of low threshold mechanoreceptors Section 2.2). In this review, we will follow a mechanistic approach, and will consider any increase in punctate mechanical pinprick sensitivity that develops at the skin surrounding the site at which intense and/or repeated nociceptive stimulation was applied as the same phenomenon as SHA.

### 3.1 Heat sensitivity in the area of SHA

**FIGURE 2:**
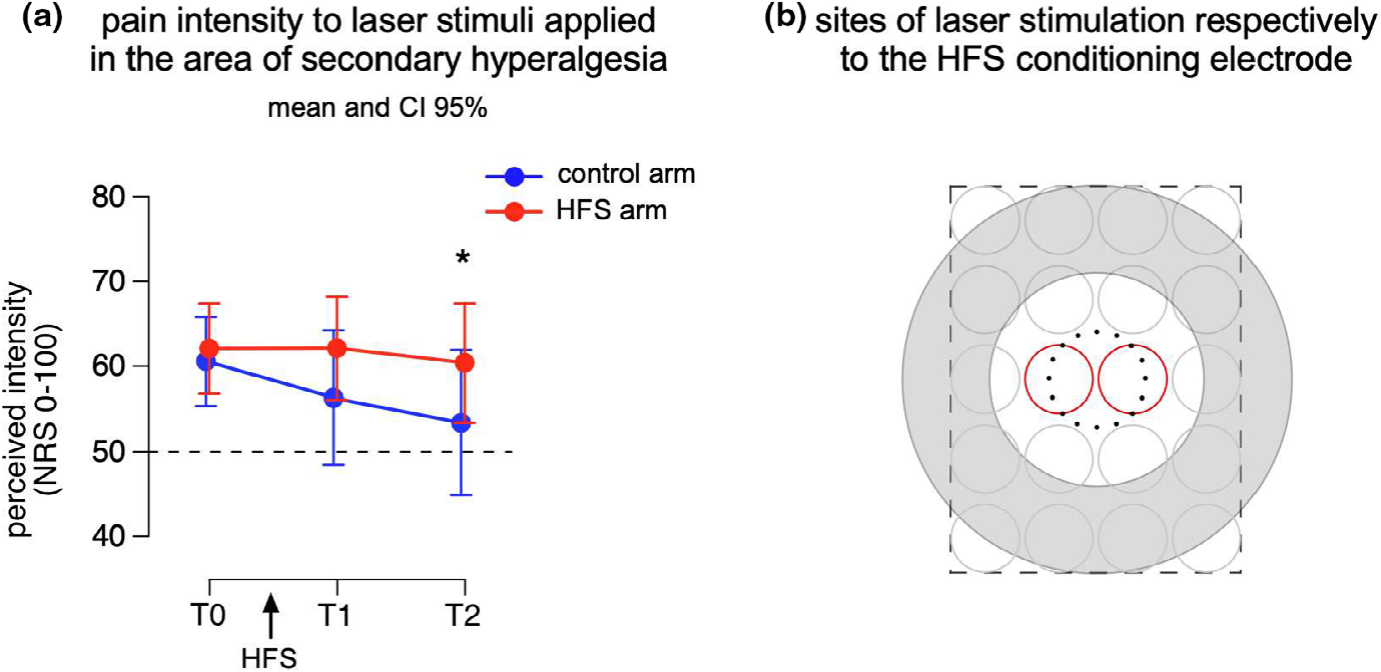
(a) Pain intensity to laser (Nd‐YAP) pulses delivered in the area of secondary hyperalgesia before (T0), 20 and 45 min after high‐frequency stimulation (HFS, 20× detection threshold) of the skin was delivered. Participants used a numerical rating scale (NRS) ranging from 0 (no perception) to 100 (the maximal imaginable pain) with 50 representing the border between non‐painful and painful sensations. In red: on the volar forearm onto which HFS was applied and in blue: on the contralateral arm. Sixteen participants (10 women; aged 20–32 years; 24.5 ± 3.8 years [mean ± SD]) were asked to rate 20 stimuli whose intensity (4.0 ± 0.5 J, [mean ± SD]) was individually adjusted to be qualified as clearly painful (NRS > 50/100). (b) Predefined sites for laser stimulation according to the location where the HFS electrode was positioned. The same template was drawn on both volar forearms. Laser pulses were in a random order on the 20 possible circles with a random inter‐stimulus‐interval (3–5 s). The laser pulses delivered at the location indicated by the two red circles were withdrawn from analysis. Mean and 95% confidence intervals are displayed. The asterisk indicates a significant difference in the perceived intensity between the control and HFS arm at T2. (Unpublished data Lenoir et al., 2019).

Whether SHA is also present for heat stimuli is controversial. Table 1 summarizes studies that have assessed changes in heat pain threshold and perceived heat sensitivity to suprathreshold heat stimuli in the area of SHA. Secondary heat hyperalgesia has been mostly studied after conditioning with transcutaneous electrical stimulation, heat injury (burn and UVB) and capsaicin application (intradermal and topical) (Table 1). Regarding transcutaneous electrical stimulation, five studies used high‐frequency electrical stimulation (HFS) and one study used low‐frequency stimulation (LFS). The two studies that applied HFS or LFS with an intensity corresponding to 10 times the individual detection threshold (to a single electric pulse) did not find an increase in perceived heat sensitivity in the area of SHA. Three out of four studies that applied HFS at an intensity corresponding to 20 times the individual detection threshold found an increase in perceived heat sensitivity after HFS. Also, unpublished data reveals a change in heat sensitivity after HFS when HFS was delivered with an intensity of 20 times the detection threshold (Figure 2a). Two out of these three studies, and the unpublished data, found a higher perceived heat sensitivity at the conditioned arm compared to the control arm, but no increase compared to baseline measurement at the conditioned arm. In these studies, a decrease in heat sensitivity after HFS at the control arm was observed and attributed to habituation. Therefore, the higher heat sensitivity observed in the conditioned arm probably reflected a lack of habituation. When heat sensitivity was assessed within the same body part by comparing ratings before versus after the delivery of HFS only one study out of five showed an increase. One study (van den Broeke, Lenoir, & Mouraux, 2016), using long‐lasting heat stimuli (30 s), did not find any significant change in heat sensitivity in the area of SHA. Nevertheless, in this study using HFS, the perceived heat intensity within the first 5 s after stimulus onset was increased in the conditioned arm as compared to the control arm. None of the studies that applied transcutaneous HFS or LFS assessed heat pain thresholds in the area of SHA. Taken together, the most consistent result seems a lack of habituation in heat perception to brief radiant heat stimuli at the experimental arm after high‐intensity HFS when comparisons in heat sensitivity were performed between the sensitized arm and a control site. Notably, all these positive studies were conducted by the same group.

**TABLE 1:** Summary of studies investigating heat sensitivity in the area of secondary mechanical hyperalgesia.

Reference	Conditioning model	Conditioning intensity	Site	Sample size	Apparatus and stimulating surface	Pain threshold decrease	Ramping rate, baseline temperature	Perceived intensity increase	Intensity and duration of the test stimulus	Comparisons factors
Lenoir et al. (2018)	HFS	20× DT	Forearm	18	CO_2_ laser, 0.07 mm^2^	n.a.	n.a.	YES	10.3 ± 3.2 mJ/mm^2^	Between arms, decrease at control arm 20 and 45 min after vs. before, no change in HFS arm
van den Broeke, Lenoir, and Mouraux (2016)	HFS	20× DT	Forearm	20	CO_2_ laser, 0.28 cm^2^	n.a.	n.a.	NO	47.6°C (45–49°C), 30 s	Control vs. HFS arms, before vs. 20 min after
van den Broeke and Mouraux (2014a)	HFS	20× DT	Forearm	17	CO_2_ laser, 0.11 cm^2^	n.a.	n.a.	YES	11.4 ± 2.8 mJ/mm^2^ for 50 ms	20 min (T1) and 45 min (T2) after vs. before (T0), same site
van den Broeke and Mouraux (2014b)	HFS	20× DT	Forearm	15	CO_2_ laser, 0.28 cm^2^	n.a.	n.a.	YES	43 ± 1°C	Between arms, increase at HFS arm and decrease at control arm at 20 and 45 min after vs. before
Vo and Drummond (2016)	HFS	10× DT	Forearm	22	Metal probe, 1.77 cm^2^	n.a.	n.a.	NO	44 ± 0.2°C for 7 s	Between arms; after vs. before at HFS arm
Vo and Drummond (2014)	LFS	10× DT	Forearm	30	Metal probe, 1.77 cm^2^	n.a.	n.a.	NO	44 ± 0.2°C for 7 s	After vs. before, same site
Geber et al. (2007)	Intradermal LFS	30 mA	Forearm	10	Thermode, 7.84 cm^2^	NO	1°C/s, 32°C	n.a.	n.a.	After vs. before, same site
Scheuren et al. (2023)	Repetitive heat pain	60 stimuli at 48°C, 6 s	Forearm	20	Thermode, 9 cm^2^ Thermode, 1.2 cm^2^	NO	10°C/s, 32°C 200°C/s, 35°C	NO	60°C, 150 to 600 ms	After vs. before, experimental vs. control condition
Jürgens et al. (2014)	Repetitive heat pain	60 stimuli at 48°C, 6 s	Forearm	18	Thermode, 2.56 cm^2^	NO	1°C/s, 32°C	n.a.	48°C, 2 s	Ipsilateral vs. contralateral sites, after vs. before experimental condition
Lotsch and Ultsch (2018)	UVB	2× MED	Forearm	28	Thermode, 9 cm^2^	YES	1°C/s, 32°C	n.a.	n.a.	After vs. before, same site
Gustorff et al. (2013)	UVB	3× MED	Upper leg	22	Thermode, 2.56 cm^2^	NO	1°C/s, 32°C	n.a.	n.a.	UVB vs. control site (contralateral)
Gustorff et al. (2004)	UVB	3× MED	Upper leg	8	Thermode, 3.24 cm^2^	NO	0.8°C/s, 32°C	n.a.	max 54°C	UVB vs. control site (contralateral)
Slimani et al. (2018)	Burn	47°C, 7 min	Anterior‐medial calf	20	CO_2_ laser, 0.3 cm^2^	n.a.	35°C pre‐heated	NO	100 ms, 38–53°C	Burn vs. control group, 1 h and 24 h after burn
Pedersen and Kehlet (1998)	Burn	47°C, 7 min	Anterior lower leg	15	Thermode, 3.75 cm^2^	n.a.	n.a.	YES	Ramping from 40 to 45°C (5 s) followed by 45°C (5 s)	Burn vs. control site, 1 and 2 h after vs. baseline
Møiniche et al. (1993)	Burn	49°C, 5 min	Calf	8	Thermode, 3.75 cm^2^	YES	1°C/s, 32°C	n.a.	n.a.	After (3, 6, 24 h) vs. baseline, same site
Raja et al. (1984)	Burn	53°C, 30 s	Hand	8	CO_2_ laser, 0.56 cm^2^	n.a.	n.a.	NO	41–49°C for 3 s	After vs. before, same site
Thalhammer and LaMotte (1982)	Burn	56°C, 7 s	Forearm	4	Copper cylinder 0.8 cm^2^	YES	n.a.	YES	51°C, 5 s, 0.03 cm^2^, ISI 30 s	Before vs. after, same site, 4 individuals
Yucel et al. (2004)	Burn	47°C, 7 min	Forearm	12	Xenon lamp, 0.47 cm^2^	YES	n.a	n.a.	0.8, 1, 1.2× PainTh, 500 ms	Burn vs. contralateral control site
Kilo et al. (1994)	Freeze lesion	−28°C, 20–40 s	Upper leg, forearm, hand dorsum	24	Thermode, 4 cm^2^	YES	0.5°C/s, 30°C	n.a.	n.a.	After vs. before, same sites
Geber et al. (2007)	Capsaicin (intradermal)	50 μg	Forearm	10	Thermode, 7.84 cm^2^	NO	1°C/s, 32°C	n.a.	n.a.	After vs. before, same site
Sumikura et al. (2005)	Capsaicin (intradermal)	100 μg	Forearm	6	Xenon lamp, 0.25 cm^2^	n.a.	n.a.	YES	0.8, 1, 1.2× PainTh, 350, 500, 750 ms	After vs. before, same site
Sumikura et al. (2003)	Capsaicin (intradermal)	250 μg	Forearm	8	Thermode, 3.14 cm^2^	n.a.	n.a.	YES	1.2× PainTh, 500 ms	After vs. before, same site
Serra et al. (1998)	Capsaicin (intradermal)	100 μg, 1%	Forearm	27	Thermode, 1 cm^2^	n.a.	n.a.	YES	47°C for 5 s	After vs. before, same site
Ali et al. (1996)	Capsaicin (intradermal)	50 μg, 1%	Forearm	16	CO_2_ laser, 0.4 cm^2^	NO	1°C/s, 38°C	NO	48°C for 2 s	After vs. before, same site
LaMotte et al. (1991)	Capsaicin (intradermal)	100 μg, 1%	Forearm	40	Thermode, 0.78 cm^2^	n.a.	n.a.	YES	38°C for 5 s	After vs. before, same site
Simone and Ochoa (1991)	Capsaicin (intradermal)	100 μg, 1%	Forearm	10	Thermode, 0.36 mm^2^	NO	9°C/s, 28°C	n.a.		Capsaicin vs. contralateral control site
Yucel et al. (2004)	Capsaicin (intradermal)	50 μg, 1%	Forearm	12	Xenon lamp, 0.47 cm^2^	YES	n.a	n.a.	0.8, 1, 1.2× PainTh, 500 ms	Capsaicin vs. contralateral control site
De Schoenmacker et al. (2021)	Capsaicin (topical)	1 mL, 0.075%	Forearm	21	YAP laser, 0.2 cm^2^ thermode, 5.7 cm^2^	n.a. n.a.	n.a. 70°C/s, 35°C	NO NO	2.85 J, 5 mm, 5 ms 52°C	Before vs. after, sham vs. capsaicin condition, same site
Hughes et al. (2020)	Capsaicin (topical)	1%	Lateral calf	14	Thermode 2.56 or 9 cm^2^	NO	32°C, 1°C/s	NO		After vs. before, same site
Lotsch and Ultsch (2018)	Capsaicin (topical)	Cream 150 mg, 0.2%	Forearm	78	Thermode, 9 cm^2^	YES	1°C/s, 32°C	n.a.	n.a.	After vs. before, same site
Hullemann et al. (2015)	Capsaicin (topical)	1 mL, 0.6%	Hand dorsum	15	Thermode, 9 cm^2^ YAP laser, 0.2 cm^2^	YES n.a.	1°C/s, 32°C n.a.	n.a. YES	n.a. 191.9 ± 30.2 mJ/mm^2^, 5 ms	After vs. before, same site
Drummond and Blockey (2009)	Capsaicin (topical)	0.02 M 0.6%	Hand	27	Radiant heat lamp	NO	0.5°C/s, 30°C	n.a.	30 to 48°C, 0.5°C/s	After vs. before, same site
Valeriani et al. (2005)	Capsaicin (topical)	1 mL, 3%^a^	Upper lip	10	CO_2_ laser, 0.25 cm^2^	n.a.	n.a.	NO	4.5 W, 30 ms	After vs. before, same site
Valeriani et al. (2003)	Capsaicin (topical)	1 mL, 3%^a^	Hand dorsum	10	CO_2_ laser, 0.03 cm^2^	n.a.	n.a.	NO	11.1 ± 2.1 (right), 10.8 ± 1.9 (left) W, 10 ms	After vs. before, same site
Yucel et al. (2002)	Capsaicin (topical)	1.5 g, 1%	Forearm	13	Thermode, 4 cm^2^	n.a.	n.a.	YES	45, 47, 49°C for 1.5 s (for 1, 5, 8°C/s ramps)	After vs. before, same site
Arendt‐Nielsen et al. (1996)	Capsaicin (topical)	1%^b^	Lower leg	10	Thermode, 3 cm^2^ argon laser, 0.07 cm^2^	NO YES	1°C/s, 30°C single pulses, 200 msec	n.a. YES	n.a. 0.8, 1, 1.2, 1.4× PainTh, 200 ms	After vs. before, same site
Andersen et al. (1996)	Capsaicin (topical)	1%, 6.25 cm^2^	Foot dorsum	17	Thermode, 3.84 cm^2^	NO	1°C/s, 35°C	n.a.	n.a.	After vs. before, same site

*Note*: Table summarizing the results of studies investigating heat sensitivity in the area of secondary hyperalgesia. Secondary hyperalgesia was induced by means of different conditioning models: HFS (high‐frequency stimulation of the skin), LFS (low‐frequency stimulation of the skin), UVB (ultraviolet‐B irradiation), burn, intradermal injection or topical application of capsaicin. To assess if heat sensitivity was enhanced in the area of secondary hyperalgesia, we report here if the heat threshold was decreased and/or if the perceived intensity for suprathreshold heat stimuli was enhanced by writing YES or NO in the corresponding columns. In the last column on the right part of the table, we detailed what were the compared conditions used to respond to this question, e.g., sensitized versus control body sites or before versus after sensitization on the same body site. Results are reported when significant effects or differences have been reported in the corresponding studies.

Abbreviations: DT, detection threshold; MED, minimal erythema dose of ultraviolet‐B; PainTh, pain threshold.

^a^ The percentage refers to the concentration of the capsicum oleoresin which corresponds to 0.3% capsaicin concentration.

^b^ The report does not mention the nature of the capsaicin solution or cream applied for 1 h.

Regarding the studies that used capsaicin, eight used intradermal capsaicin injection and 10 applied capsaicin topically. Among the studies that applied capsaicin intradermally four studies investigated heat pain thresholds: three did not observe any significant change and one observed a decrease. Five studies assessed if there was a change in heat perception and four of those found an increase. Of the 10 studies that applied capsaicin topically six assessed changes in pain thresholds including one in which both contact and radiant heat thresholds were measured. Three experiments found a decrease in pain threshold and four experiments did not find any change. Change in heat sensitivity was assessed in eight different experimental settings among seven out of 10 studies using topical capsaicin. Among them only three studies found an increase. Taken together, changes in perceived heat sensitivity seem most prominent after intradermal capsaicin injection and suprathreshold contact heat stimuli.

The results regarding repetitive heat pain, burn injury or UVB conditioning methods are more inconsistent. Five out of eight studies that used heat pain or burn assessed changes in pain thresholds. Three of these found a decrease in threshold and two found no change. Five of the studies that used heat pain or burn assessed changes in perceived heat sensitivity. Two found an increase and three did not. Three studies that used UVB assessed pain thresholds. Two studies did not observe any change while one did. None of the UVB studies assessed changes in perceived heat sensitivity.

In summary, the perceived intensity elicited by heat stimuli is not consistently changed in the area of SHA. When present, it involves mainly an increase in perceived intensity to suprathreshold stimuli rather than a change in heat pain threshold. Changes in heat sensitivity are mostly reported following intradermal capsaicin injection or transcutaneous HFS. Changes in heat sensitivity are noted particularly for brief radiant heat stimuli (delivered by infra‐red laser stimulators) after HFS and for brief contact heat stimuli (delivered by the thermode) after intradermal capsaicin injection. This pattern is likely biased by the frequent use of laser stimuli in HFS studies and contact heat in capsaicin studies. Overall, the changes in heat sensitivity in the area of SHA involve a lack of habituation at the sensitized site as compared to the control site rather than an increase compared to the baseline.

### 3.2 Tactile mechanical sensitivity in the area of secondary hyperalgesia

**FIGURE 3:**
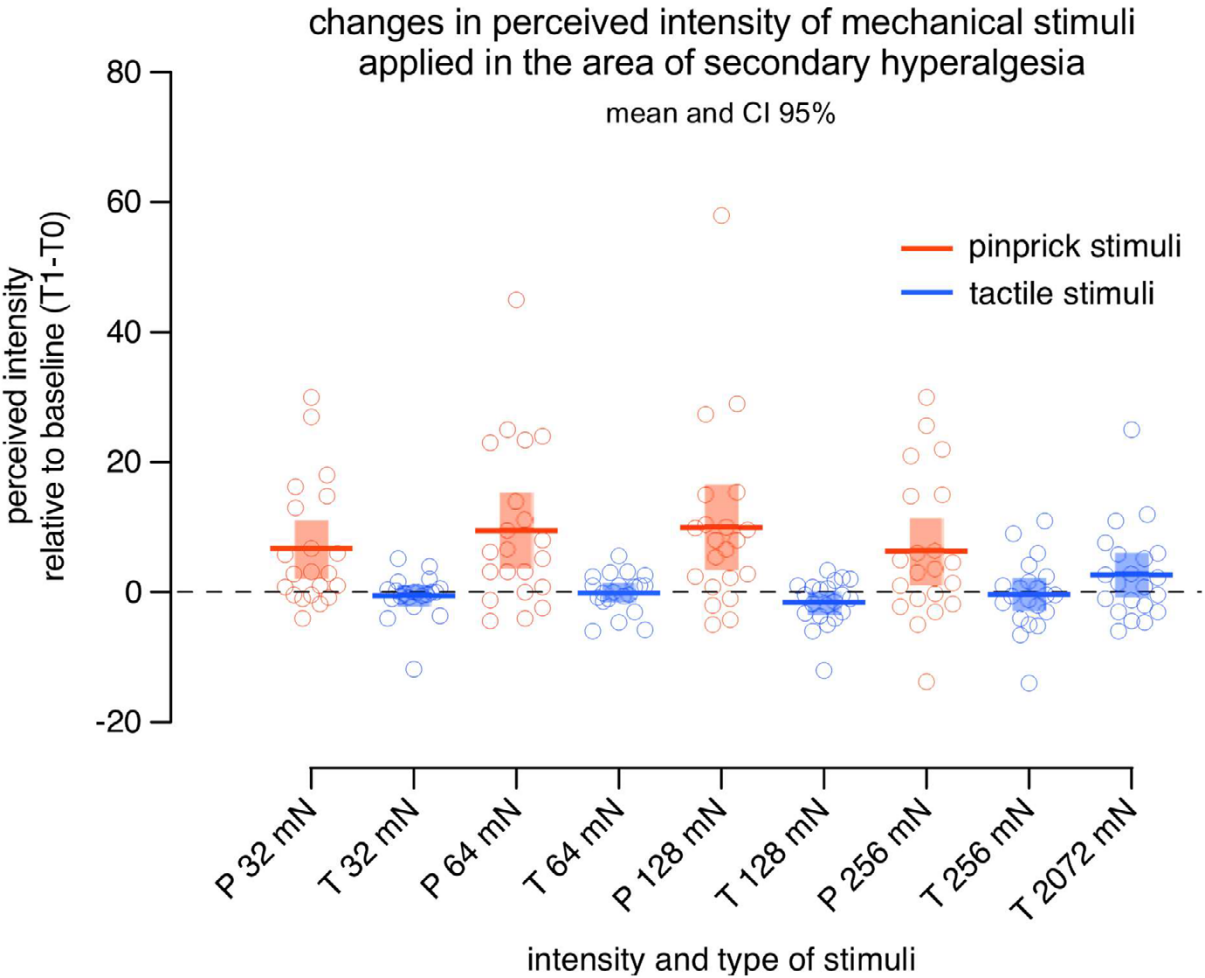
Increased perceived intensity for different pinprick mechanical stimuli and not for tactile stimuli delivered in the area of secondary hyperalgesia induced by HFS. The change in perceived intensity was computed by subtracting the ratings obtained before HFS (baseline T0) from the ratings obtained 20 min after HFS (T1). Participants used a numerical rating scale (NRS) ranging from 0 (no perception) to 100 (the maximal imaginable sensation) and additionally provided descriptors for the quality of the sensation and if the sensation was painful or not. Pinprick stimuli with a blunt tip (P; in red; diameter 0.35 mm) and tactile stimuli with a round tip (T; in blue; diameter 2 mm) of four intensities (32, 64, 128 and 256 mN) were delivered in a counterbalanced manner onto the skin surrounding the site where the HFS electrode was positioned. In addition, a tactile stimulator with a round tip of 2072 mN which matched the pressure elicited by the pinprick 64 mN was also used. Mean and 95% confidence intervals are displayed. Across all intensities, the perception of pinprick stimuli (in red) was enhanced after HFS which was not the case with the tactile stimuli (in blue) of the same intensities or stimuli exerting the same pressure when applied onto the skin. (21 participants; 11 women; aged 18–29 years; 21.1 ± 2.7 years [mean ± SD]). (Unpublished data Lenoir et al., 2021).

**FIGURE 4:**
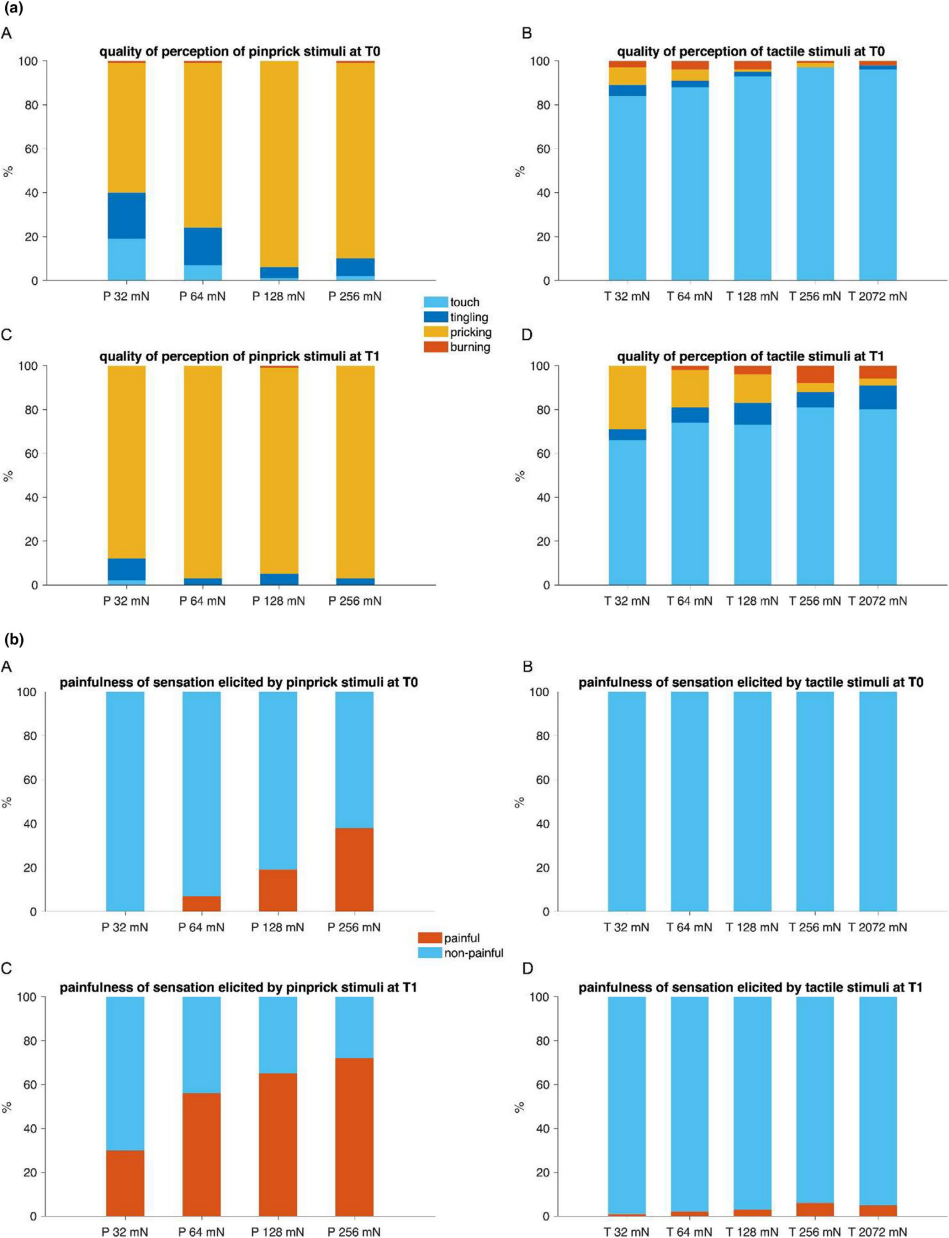
(a) Proportion of each descriptor used to describe the quality of perception of the pinprick and tactile stimuli delivered in the area of secondary hyperalgesia before (T0) and after (T1) HFS. (a, c) Pinprick stimuli (P; stimulator with blunt tip; diameter 0.35 mm) and (b, d) tactile stimuli (T; stimulator with round tip; diameter 2 mm) of four intensities (32, 64, 128 and 256 mN) were delivered in a counter‐balanced manner onto the skin surrounding the site where the HFS electrode was positioned. In addition, a tactile stimulator of 2072 mN which matched the pressure elicited by the pinprick 64 mN was also used. Descriptors considered to qualify are displayed in light blue for ‘touch’, dark blue for ‘tingling’, in orange for ‘pricking’ and red for ‘burning’. When applied to sensitized skin the quality of pinprick stimuli and to a lesser extent of tactile stimuli was changed with pinprick stimuli more often perceived as ‘pricking’ and tactile ones more often perceived as ‘pricking’ and ‘burning’. (21 participants; 11 women; aged 18–29 years; 21.1 ± 2.7 years [mean ± SD]). (Unpublished data Lenoir, van den Broeke et al., 2021). (b) Proportion of stimuli perceived as ‘painful’ and ‘non‐painful’ before (T0) and after (T1) HFS. (a, c) Pinprick stimuli (P; stimulator with blunt tip; diameter 0.35 mm) and (b, d) tactile stimuli (T; stimulator with round tip; diameter 2 mm) of four intensities (32, 64, 128 and 256 mN) were delivered in a counter‐balanced manner onto the skin surrounding the site where the HFS electrode was positioned. In addition, a tactile stimulator of 2072 mN which matched the pressure elicited by the pinprick 64 mN was also used. Proportions of stimuli perceived as ‘painful’ and ‘non‐painful’ are displayed respectively in blue and red. When applied to sensitized skin pinprick stimuli of different intensities are in 30%–70% of the cases perceived as ‘painful’ which is the case for only 6% maximum of the cases for tactile stimuli. The increase of pinprick stimuli perceived as painful after HFS is clearly visible which is not the case for tactile stimuli (21 participants; 11 women; aged 18–29 years; 21.1 ± 2.7 years [mean ± SD]). (Unpublished data Lenoir et al., 2021).

SHA clearly manifests itself by the change in perception of sharp punctate mechanical stimuli, such as pinprick stimuli. However, mechanical indenting stimuli (with diameter in the 0.5 mm range and of intensity dozens of mN) which activate high‐threshold mechano‐nociceptors (HTMs) unavoidably activate also low‐threshold mechanoreceptors (LTMs) and notably slowly and rapidly adapting type I mechanoreceptors (Cohen & Vierck, 1993; Johansson & Vallbo, 1979; Vallbo & Johansson, 1984). This raises the question if there is a contribution of LTMs to the increase in mechanical sensitivity elicited by static indenting stimuli. Table 2 summarizes studies that have assessed the involvement of tactile mechanical sensitivity in the area of SHA. Figure 3 shows the results of an unpublished study in which tactile stimuli and pinprick stimuli of the same intensity were applied to the area of SHA induced by HFS. In that study, no increase in the perceived intensity elicited by the tactile stimuli were found. In contrast, the perception of mechanical pinprick stimuli was clearly increased. These results, thus, suggest that LTMs, activated by tactile stimuli of different intensities do not contribute significantly to SHA. In the same line, Vollert et al. (2018) found no evidence for changes in both mechanical detection thresholds using rounded tip punctate stimuli or vibrations in the area of SHA. One study showing a change in the perception of tactile stimuli specifically using modified von Frey filaments with round tips reported those changes for stimuli delivered at intensities higher than 32 mN which might increase the probability of recruiting HTMs concomitantly to LTMs. Indeed, an increase in sensations qualified as pricking and painful can be observed for tactile stimuli delivered at high intensities after HFS (Figure 4a,b). Another study reported a decrease in the threshold for vibration using topical capsaicin in two different concentrations (0.005% and 0.01%) (Andrews et al., 1999). This study included only six participants and explored vibration using a vibration stimulator applied with a concomitant pressure (650 g) in addition to the vibrating stimulus, therefore likely affecting deep tissues and not comparable with other studies.

**TABLE 2:** Summary of studies investigating tactile sensitivity in the area of secondary mechanical hyperalgesia.

Reference first author, year	Conditioning model	Conditioning intensity	Site	Sample size	Apparatus and stimulating surface	Threshold decrease	Intensity, surface of stimulus	Perceived intensity increase	Intensity, duration, surface of stimulus	Comparisons factors
Vollert et al. (2018)	Capsaicin (intradermal)	100 μg, 10 μL	Forearm	36	Modified von Frey filaments (round tips) tuning fork	NO NO^b^	0.25 to 512 mN, 0.5 mm^d^ 64 Hz, 8/8 scale^d^	n.a. n.a.	n.a. n.a.	Capsaicin vs. control site (contralateral)
van den Broeke et al. (2014)	HFS	20× DT	Forearm	17	Mechanical transducer	n.a.	n.a.	NO	300 Hz,50 ms, 4 cm^2^	20 min (T1) and 45 min (T2) after vs. before (T0), between HFS and control arms
Gustorff et al. (2013)	UVB	3× MED	Upper leg	22	Modified von Frey filaments (round tips) tuning fork	NO NO	0.25 to 512 mN, 0.5 mm^d^ 64 Hz, 8/8 scale^d^	n.a. n.a.	n.a. n.a.	UVB vs. control site (contralateral)
De Col and Maihöfner (2008)	Trancutaneous electrical stimulation^a^	Pain NRS 5/10	Forearm	50	Modified von Frey filaments (round tips)	NO^b^	0.25 to 512 mN, 0.5 mm^d^	n.a.	n.a.	After vs. before
Stammler et al. (2008)	Trancutaneous electrical stimulation^a^	Pain NRS 5/10	Forearm	12	Modified von Frey filaments (round tips)	NO^b^	0.25 to 512 mN, 0.5 mm^d^	n.a.	n.a.	After vs. before
Magerl et al. (1998)	Capsaicin (intradermal)	40 μg, 12.5 μL	Forearm	12	Modified von Frey filaments (round tips)	YES^c^	0.5 to 4100 mN, 1.1 mm	YES	256 mN, 1.1 mm	After vs. before, capsaicin vs. control site
Andrews et al. (1999)	Capsaicin (topical)	0.05 mg/mL 0.005%	Forearm	6	Vibration	YES	1 cm 100 Hz	n.a.	n.a.	Capsaicine vs. control
Andrews et al. (1999)	Capsaicin (topical)	0.1 mg/mL 0.01%	Forearm	6	Vibration	YES	1 cm 100 Hz	n.a.	n.a.	Capsaicine vs. control

*Note*: Table summarizing the results of studies investigating static tactile sensitivity in the area of secondary hyperalgesia. Secondary hyperalgesia was induced by means of different conditioning models: HFS (High‐frequency stimulation of the skin), UVB (ultraviolet‐B irradiation), transcutaneous electrical stimulation (35 min at different frequencies and stimulation patterns), intradermal injection or topical application of capsaicin. Similar conventions as in table heat (to assess if static tactile sensitivity was enhanced in the area of secondary hyperalgesia, we report here if the threshold was decreased and/or if perceived intensity for suprathreshold heat stimuli was enhanced by writing YES or NO in the corresponding columns. In the last column on the right part of the table, we detailed what were the compared conditions used to respond to this question, for example, between sensitized and control body sites or before versus after sensitization on the same body site). Results are reported when significant effects or differences have been reported in the corresponding studies.

Abbreviations: DT, detection threshold; MED, minimal erythema dose of ultraviolet‐B.

^a^ See references for conditioning stimulation parameters.

^b^ A significant increase in the tactile detection threshold was observed.

^c^ No change in perceived intensity up to 32 mN, and no change in pain incidence up to 8 mN.

^d^ DFNS standardized protocol (see Rolke et al., 2006).

### 3.3 Can we detect secondary hyperalgesia using electrical stimuli?

Unlike thermal and mechanical, electrical stimuli bypass peripheral receptor transduction and activate directly voltage‐gated ion channels. It should be stressed that the characteristics of the electrical test stimuli, such as the electrode type, the stimulation intensity and the pattern of stimulation are crucial to establish the relative selectivity of the electrical stimulus. In other words, those parameters will determine how preferentially the stimulus will recruit nociceptive versus non‐nociceptive nerve fibre (La Cesa et al., 2018; Legrain & Mouraux, 2013; Mouraux et al., 2010, 2014; Poulsen et al., 2022).

Transcutaneous electrical stimulation through surface electrodes elicits an electric current that propagates deep in the dermis. Depending on the intensity, this stimulation can activate preferentially large diameter non‐nociceptive fibre in association with thinly myelinated nociceptive fibres. Such stimulation has been used in three studies. In the first study (van den Broeke et al., 2010) the authors did not find any significant increase in the perceived intensity elicited by transcutaneous electrical stimuli in the area of SHA induced by HFS as compared to baseline, for electrical stimulation delivered at an intensity below the pain threshold (50% of pain threshold intensity). However, the authors reported a significant difference in the perceived intensity after HFS between the HFS arm and the control arm. The authors concluded that sensations mediated by A‐delta fibres might be affected (less habituation) in sensitized skin. Yucel et al. (2004) used transcutaneous electrical stimulation delivered with surface electrodes to assess changes in pain thresholds after SHA was induced using both intradermal capsaicin and a heat injury model. They found a decreased electrical pain threshold in both experimental methods using a pulse train of five pulses of 1 ms delivered at 200 Hz which was perceived as a single stimulus and was intended to mainly recruit small, myelinated fibres. More recently, Hughes et al. (2020) used transcutaneous electrical stimuli delivered with surface electrodes to assess changes in pain thresholds after SHA was induced using topical capsaicin application. They found lowered pain thresholds for electrical stimuli delivered in the area of SHA. Interestingly, the authors observed that the magnitude of hyperalgesia to electrical stimuli was far from being uniformly distributed within the area of SHA. In this study, it is likely that the electrical stimulation delivered at a high intensity (approximately 18 mA) and by means of surface electrodes recruited small and large diameter fibres, it is therefore difficult to conclude about the type of afference responsible for the observed perceptual increase to electrical stimuli delivered in the area of SHA.

A multipin electrode designed to preferentially activate cutaneous free nerve endings and used for conditioning stimulation in HFS protocols (Klein et al., 2004) has been used recently to investigate pain perception elicited by painful single electrical stimuli (intensity approximately 1.7 mA) in the area of SHA induced by HFS (van den Broeke et al., 2021). The authors reported an overall significant decrease in pain perception to electrical stimuli after HFS at both the control and HFS sites. Although pain ratings were globally significantly decreased after HFS, the participants reported significantly higher pain ratings for stimuli delivered to the area of SHA as compared to the control site. This study showed again that perception of electrical stimuli was affected in sensitized versus control sites, similarly as in van den Broeke et al. (2010).

Two studies used low‐intensity intra‐epidermal electrical stimulation (IES, Inui et al., 2002) to investigate the contribution of A‐delta fibre nociceptors to SHA (Biurrun Manresa et al., 2018; Liang et al., 2016). Liang et al. (2016) showed that the intensity of perception elicited by IES (intensity at twice the detection threshold ~0.5 mA (personal communication)) was significantly increased in participants who developed ‘robust SHA’ (referred to as ‘responders’) after intradermal capsaicin injection. However, using HFS to induce SHA, Biurrun Manresa et al. (2018) did not find evidence for an increase in the perceived intensity of these stimuli (intensity at twice the detection threshold ~0.14 mA) in the area of SHA. These apparently contradictory results might be due to the different methods used to induce SHA, that is, intradermal capsaicin injection versus HFS, and/or to the difference in IES intensity, which was considerably higher in the study of Liang et al. (2016). Regarding this latter, Legrain and Mouraux (2013) have shown that with higher stimulation intensities, intra‐epidermal electrical stimulation loses its selectivity for activating nociceptive afferents.

## 4 ELECTROPHYSIOLOGICAL MEASURES

### 4.1 Spinal cord activity assessed by the nociceptive flexion reflex

The nociceptive flexion reflex (NFR) is a polysynaptic and multi‐segmental spinal reflex that induces a complex flexion synergy of the stimulated limb, whose anatomical substrate is entirely located at the spinal level (Sandrini et al., 2005). It can be elicited by transcutaneous electrical stimulation of either the upper or lower limb, however, it has been mainly investigated in the lower limb. The withdrawal reflex consists of two separate components, namely the RII component generated by the activation of large‐myelinated fibres (Hugon, 1973) and the RIII component elicited at higher intensities due to the additional recruitment of thinly myelinated fibres (Ertekin et al., 1975). Due to the close correspondence existing between the RIII reflex threshold and the subjective pain threshold (Willer et al., 1984), this component has been considered by some researchers as an ‘objective’ measure of experimental pain in humans (Sandrini et al., 2005). The recording of the RIII is performed using an electromyogram (EMG) recording device. Several protocols have been proposed to induce the reflex, the more common setup being the stimulation of the peripheral nerve with a train of five 1‐ms electrical square pulses delivered at a frequency of 200 Hz. The reflex response is obtained from the recording of EMG activity of the flexor muscle using surface electrodes. The reflex assessment foresees first the estimation of the threshold defined as the intensity evoking a stable response at a rate of 50–60% after 20–30 stimuli, using a staircase method (Andersen et al., 2005; Sandrini et al., 1993). The reflex recording is then performed using a stimulation intensity multiple of that of the threshold. Several parameters of the RIII can be assessed, including threshold, amplitude, latency, receptive field, temporal summation, response rate and habituation (Andersen et al., 1995; Arendt‐Nielsen et al., 1994; Biurrun Manresa et al., 2014; Dimitrijević et al., 1972; Willer, 1977). Several studies explored the capability of the RIII to detect spinal excitability changes induced by different experimental methods to induce SHA, which are summarized in Table 3. Nearly all the studies explored the lower limb RIII reflex after stimulation of either the tibial or the peroneal nerve, except the study by Linde et al. (2021), where the authors explored the upper limb after stimulation of the index finger.

**TABLE 3:** Summary of studies investigating NFR modulation by secondary hyperalgesia models.

Reference	Conditioning stimulus	Ongoing pain	SHA testing	Site	Sample size	Intensity and duration of test stimulus	Recording site	Pain rating	Reflex thr↓	Reflex AUC ↑	Comparison factors
Grönroos and Pertovaara (1993)	Capsaicine 1% (topical)	YES	YES^a^	Foot dorsum	7	Sural: train of 4 pulses (1 ms duration) delivered over 40 ms	Ipsilateral biceps femoris	n.a	YES	n.a	Before vs. 45 min after H reflex
Andersen et al. (1995)	Capsaicine 0.1% (topical)	NO	NO	Foot dorsum	8	Sural: train of 5 pulses (1 ms duration), 200 Hz	Ipsilateral biceps femoris/rectus femoris	NO	n.a	NO	Before vs. 60 min after H reflex
	Capsaicine 0.1% (topical) + Aβ activity in SHA area (40 Hz electrical stimulation/rotating V.Freys)	NO	NO	Foot dorsum	8	Sural: train of 5 pulses (1 ms duration), 200 Hz	Ipsilateral biceps femoris/rectus femoris	NO	n.a	NO	Before vs. 60 min after H reflex
	Capsaicine 0.1% (topical) + continuous heat (38°C) in PHA	YES	NO	Foo t dorsum	8	Sural: train of 5 pulses (1 ms duration), 200 Hz	Ipsilateral biceps femoris /rectus femoris	YES	n.a	YES	Before vs. 60 min after H reflex
	Capsaicine 0.1% (topical) + heat (38°C, 30 s) +40 Hz electrical stimulation (in SHA area)	YES	NO	Foot dorsum	8	Sural: train of 5 pulses (1 ms duration), 200 Hz	Ipsilateral biceps femoris/rectus femoris	YES	n.a	YES	Before vs. 60 min after H reflex
Andersen et al. (1996)	Capsaicine 1% (topical)	n.a.	YES	Foot dorsum	17	Sural: train of 5 pulses (1 ms duration), 200 Hz	Ipsilateral biceps femoris/rectus femoris	NO	n.a	NO	Before vs. 60 min after Placebo vs. ketamine
	Capsaicine 1% (topical) + heat (32–40°C)	YES	YES	Foot dorsum	17	Sural: train of 5 pulses (1 ms duration), 200 Hz	Ipsilateral biceps femoris/rectus femoris	YES	n.a	YES	Before vs. 60 min after placebo vs. ketamine
	Capsaicine 1% (topical) + heat (32–40°C) + 40 Hz electrical stimulation (in SHA area)	YES	YES	Foot dorsum	17	Sural: train of 5 pulses (1 ms duration), 200 Hz	Ipsilateral biceps femoris/rectus femoris	NO	n.a	YES	Before vs. 60 min after Placebo vs. ketamine
Biurrun Manresa et al. (2014)	Capsaicine 0.1% (intradermal)	YES	NO	Flexor digitorum brevis muscle	14	Foot sole: train of 5 pulses (1 ms duration), 200 Hz	Tibialis anterior	n.a	YES	n.a	Before vs. 60 min after
Leone et al. (2021)	Capsaicine 0.1% (topical)	NO	YES	Foot dorsum	12	Sural: train of 5 pulses (1 ms duration), 200 Hz	Ipsilateral biceps femoris	YES	YES	NO	Before vs. after Active vs. control
Linde et al. (2021)	Capsaicine 0.075% (topical)	YES	YES^a^	Lateral forearm	20	Palmar index finger: train of 5 pulses (1 ms duration), 200 Hz	Flexor carpi ulnaris	n.a	YES	n.a	Before vs. after Active vs. control
Ellrich and Treede (1998)	Tonic Heat (from 32 to 46°C 90 s× 8 temperatures)	YES	NO	Lateral lower leg	11	Medial Plantar: train of 5 pulses (0.1 ms duration), 200 Hz	Ipsilateral tibialis anterior	n.a	n.a	YES	Before vs. after
Guekos et al. (2023)	Tonic Heat (from 32 to 46°C 90 s× 8 temperatures)	YES	NO	Lateral lower leg	16	Sural: train of 5 pulses (1 ms duration), 200 Hz	Ipsilateral biceps femoris	NO	n.a	NO	Before vs. after
Leone et al. (2021)	HFS (5 trains, 100 Hz, 20× dT)	NO	YES	Foot dorsum	14	Sural: train of 5 pulses (1 ms duration), 200 Hz	Ipsilateral biceps femoris	YES	YES	NO	Before vs. after Active vs. control
Biurrun Manresa et al. (2010)	HFS (5 trains, 100 Hz, 10× dT)	NO	YES	Foot dorsum	13	Medial dorsal cutaneous branch of the superficial peroneal nerve: train of 5 pulses (1 ms duration), 200 Hz	Ipsilateral biceps femoris	NO	n.a	NO	Before vs. after (each 10 min, 7 times) Maximum response before vs. after 10 min
Biurrun Manresa et al. (2010)	LFS (1 train, 1000 pulses, 10× dT)	NO	YES	Foot dorsum	13	Medial dorsal cutaneous branch of the superficial peroneal nerve: train of 5 pulses (1 ms duration), 200 Hz	Ipsilateral biceps femoris	NO	n.a	YES	Before vs. after (Each 10 min, 7 times) Maximum response before vs. after 10 min

*Note*: Results are reported when significant effects or differences have been reported in the corresponding studies.

^a^ The authors only tested the presence of allodynia to light tactile stimulus.

#### 4.1.1 Reflex threshold and pain threshold modulation

When tested, the reflex threshold at the sensitized side exhibited a decrease in each of the five experimental settings reported in four studies, regardless of the experimental method used to induce SHA (capsaicin or HFS) and the considered comparisons, that is, before versus after the conditioning paradigm (Biurrun Manresa et al., 2014; Grönroos & Pertovaara, 1993; Leone et al., 2021; Linde et al., 2021) or sensitized versus control side (Leone et al., 2021; Linde et al., 2021). Based on the consistency of the results, the reflex threshold can be considered a reliable proxy of spinal excitability changes that occur in a sensitized state induced by different methods of secondary hyperalgesia. Only one study Leone et al. (2021) reported data on pain thresholds, that is, the lowest stimulation intensities causing a painful sensation, and reflex thresholds together, and as expected (Willer, 1977) found a correlation between the two measures (*r* = 0.738; *p* < 0.0001, unpublished results).

#### 4.1.2 Modulation of the reflex area under the curve (AUC)

The EMG activity resulting in the flexor muscle after the nociceptive stimulation can be quantified using the reflex AUC after the signal of a selected interval of interest being rectified. The assessment of reflex AUC changes in humans, using different experimental methods to induce SHA, provided conflicting results. Some authors were able to detect an increase in the AUC only when an ongoing continuous afferent input activating nociceptive primary afferents was provided to the spinal cord concomitantly to the recording of the reflex. Andersen et al. (1995) showed that topical application of capsaicin (0.1%) alone did not positively modulate the reflex AUC. However, when a concomitant nociceptive input was delivered in the area of primary hyperalgesia evoking an ongoing pain sensation, a facilitation of the reflex was observed (Table 3). The authors replicated their results in a subsequent study (Andersen et al., 1996). Ellrich and Treede (1998) described an increased AUC after the induction of SHA by tonic heat stimulation, eliciting an ongoing painful sensation. However, this observation was not confirmed in a recent replication study using tonic heat to induce sensitization (Guekos et al., 2023). It should be stressed that in this latter study the induction of SHA was not tested by the presence of mechanical hyperalgesia in the area surrounding the conditioning site. The authors could not detect any change in pain ratings and concluded that the conditioning method was likely unsuccessful in affecting spinal excitability. Biurrun Manresa et al. (2010) provided evidence of facilitation of the reflex in terms of increased AUC after the induction of SHA by LFS (Biurrun Manresa et al., 2010). Of note, the authors detected an increased AUC after the cessation of the conditioning stimulus and therefore in the absence of any ongoing painful sensation. All the other studies, exploring a modulation of the reflex AUC after the induction of SHA with different methods of sensitization, either topical capsaicin alone or HFS, did not find any modulation of the reflex AUC after inducing sensitization (Table 3). The heterogeneity of the experimental settings and methods used to calculate the area (root mean square vs. integral of the rectified EMG, non‐standardized windows of interest), may account for the discrepancies between studies and prevent drawing conclusions on the ability of this parameter to detect the spinal excitability changes induced by different methods of sensitization. In all but one study (Biurrun Manresa et al., 2010), the reflex AUC is modulated when the reflex is tested during an ongoing pain sensation produced by the sensitization procedure. The authors also observed a modulation of the AUC after the sensitization procedure (LFS) without any ongoing pain sensation. A possible explanation could be that LFS provided a prolonged peripheral nociceptive input likely mimicking peripheral activity present during pain. Two studies reported information on a possible relationship between the increase in perceived intensity elicited by pinprick stimuli in the area of SHA and the changes in the reflex threshold or AUC. Leone et al. (2021) found no correlation between the hyperalgesia score and the decrease in the reflex threshold neither for capsaicin nor for HFS (unpublished data). Biurrun Manresa et al. (2010) tested the time course of both pinprick perception and reflex AUC every 10 min, from 20 min before LFS to 60 min after. Though a correlation analysis has not been performed, the authors showed that the two variables behaved differently.

Several studies provided data on test–retest reliability for the RIII threshold (Micalos et al., 2009; Lewis et al., 2012; Rhudy & France, 2011) describing a between session ICC of 0.72 and 0.82, and a test–retest correlation coefficient of 0.83, respectively. One study reported data on the reliability of the RIII pain rating, describing a between session ICC of 0.88 (Lewis et al., 2012). No data are available so far on the test–retest reliability of the RIII variables modulation during pain methods induction.

### 4.2 Spinal cord activity assessed by spinal somatosensory evoked potentials

The N13 is a spinal component of the somatosensory evoked potentials mediated by non‐nociceptive Aβ fibres, elicited by electrical stimulation of mixed nerves above the motor threshold (evoking a muscular twitch) and, best recorded at the level of the C6 spinous process referenced to the glottis (Desmedt & Cheron, 1981, 1982).

The origin of this component has long been debated (Cracco, 1973; Jones, 1977; Matthews et al., 1974), due to distortions introduced by the use of a midfrontal (Fz) scalp reference. The subsequent introduction of a non‐cephalic reference, which allowed the recording of the cervical potential without the contamination by scalp potentials (Desmedt, 1984), indicated a stable transversely oriented generator in the dorsal horn of the cervical cord near the spinal entry, compatible with the synaptic activity of collateral fibres of the ascending dorsal column axons as described in cats (Scheibel & Scheibel, 1968). These conclusions came from the evidence that the N13 reversed its polarity when recorded at the anterior aspect of the neck (Desmedt & Cheron, 1981) and remained unaffected in a patient with a spinal lesion above C4 with complete destruction of the dorsal funiculus (Mauguière & Courjon, 1981). In patients with syringomyelia, which pathology is an expanding cavity in the central spinal canal compressing the spinothalamic tract neurons that decussate in the anterior white commissure and preserving the distally located posterior columns, a reduced or absent N13 has been shown, even if it is not mediated by peripheral nociceptive fibres activation. These results suggest that the N13 might reflect the postsynaptic activity of cells receiving, at least in part, spinothalamic tract afferent input (Restuccia & Mauguière, 1991). The properties of the N13 in humans are very similar to those of the ‘intermediary cord potentials’ in animals. Gasser and Graham (1933) first described a series of slow potentials that could be recorded from the surface of the spinal cord in response to stimulation of dorsal roots which are thought to reflect the activation of interneurons in lamina IV and V of the dorsal horn in response to large‐myelinated fibres input (Beall et al., 1977). Thanks to the evidence described above and the similarities with animals' spinal potentials, the N13 has been recently reconsidered and proposed as a possible readout of dorsal horn excitability changes occurring during central sensitization. Di Lionardo et al. (2021) demonstrated that spinal excitability changes triggered by topical capsaicin (0.1%) application and testified by the presence of an area of secondary hyperalgesia, increased the amplitude of the N13 component in 10 healthy participants. Furthermore, with a double‐blind placebo‐controlled crossover design, the authors showed that pregabalin, a drug with proven efficacy at the dorsal horn level (Tuchman et al., 2010), was able to prevent the increase in the N13 amplitude. A subsequent study by the same group (Di Pietro et al., 2021) demonstrated a negative modulation of the N13 component amplitude by a heterotopic noxious conditioning stimulation paradigm. The amplitude of the N13 was tested before, during and after a cold pressor test. The ANOVA on the amplitude of the N13 showed a significant effect of time across the three time points disclosing an N13 amplitude reduction by 25% during the cold pressor test as compared with before. Since the heterotopic noxious conditioning stimulation simultaneously modulated pain processing remotely, as tested using pressure pain threshold, plausibly affecting wide dynamic range (WDR) neuron excitability (Leone & Truini, 2019), the authors concluded that the N13 negative modulation could be explained by a modulation of WDR neurons' activity and, therefore, suggested WDR as a possible generator of the N13.

Recent research tested a modulation of the N13 component by different experimental pain methods inducing SHA, namely low‐ and high‐frequency electrical stimulation (LFS and HFS) (Leone et al., 2023). The authors found an increased amplitude of the N13 after LFS, on both active and control sides but no effect of HFS. The authors interpreted their results as LFS and HFS being able to trigger central sensitization through different mechanisms: thanks to its peculiar stimulation parameters LFS may induce dorsal horn excitability changes through two distinct mechanisms, namely wind‐up and heterotopic facilitation, while HFS may be able to induce heterotopic facilitation only.

Taken together, these albeit limited evidence, suggest WDR neurons as possible generators of the N13 component of somatosensory evoked potential, which in turn could be a useful readout of dorsal horn excitability changes occurring during central sensitization. However, the ability to detect a modulation of the N13 seems to depend on the experimental pain method used to induce CS, possibly requiring a long‐lasting low‐frequency ongoing afferent input to the spinal level.

Test–retest reliability has been assessed in a few studies, after stimulation of the median and tibial nerve, and showed overall good reliability for amplitudes (Spearman correlation coefficient = 0.69, [84]; ICC = 0.94) (Romani et al., 1996). No data are available on the test–retest reliability of the N13 amplitude increase. No correlation has been found between the N13 amplitude increase and the magnitude of change of mechanical pinprick sensitivity after capsaicin application (*p* = 0.6; Pearson *r* = −0.14) (Di Lionardo et al., 2021).

### 4.3 Cortical activity assessed by pinprick‐evoked brain potentials

Calibrated mechanical pinprick stimuli delivered onto the skin elicit pinprick‐evoked brain potentials (PEPs) in the electroencephalogram (EEG). Evoked potentials are voltage polarity fluctuations time‐locked to the onset of a stimulus. It is yet unclear what is the functional significance of PEPs.

PEPs consist of a biphasic wave involving a negative peak that reaches a maximum of around 100–150 ms after stimulus onset and is followed by a positive peak reaching its maximum around 250–300 ms (Iannetti et al., 2013). Both PEPs peaks are maximal at central EEG electrodes.

The latencies of PEPs are compatible with the peripheral conduction velocities of A‐fibres. Mechanical pinprick stimuli inevitably activate both A‐fibre high‐threshold mechanoreceptors (HTMs) and A‐fibre low‐threshold mechanoreceptors (LTMs, Nagi et al., 2019) which makes therefore the interpretation of PEPs ambiguous regarding mediating primary afferent population. When the same mechanical pinprick stimuli are delivered to skin that has developed increased pinprick sensitivity in response to either topical capsaicin application, HFS or heat, they elicit, depending on the study, an increase in the amplitude of either the negative (Iannetti et al., 2013; Scheuren et al., 2020) or positive peak (van den Broeke et al., 2015, 2017; van den Broeke, Lambert, et al., 2016) (Table 4).

**TABLE 4:** Summary of studies investigating amplitudes and/or latencies of pinprick evoked potentials (PEPs).

Reference	Stimulation method	Conditioning model	Body location	Sample size	Pinprick intensity (mN)	N2 amplitude increase	N2 latency (ms)	P2 amplitude increase	P2 latency (ms)	Comparison factors
Iannetti et al. (2013)	Manual	Capsaicine intradermal	Hand dorsum	10	128	YES	111 ± 8^a^	NO	245 ± 17	Ipsilateral vs. contralateral sites, after vs. before
van den Broeke et al. (2015)	Manual	Capsaicine intradermal	Ventral forearm	16	AVG	NO		YES		Ipsilateral vs. contralateral sites, after vs. before
van den Broeke, Lambert, et al. (2016)	Manual	HFS 20× DT	Forearm	16	64 90	NO NO		YES NO		Control vs. HFS arms, before vs. 20, 45 min after
van den Broeke et al. (2017)	Manual	HFS 20× DT	Forearm	16	64 96	NO NO		YES NO		Ipsilateral vs. contralateral sites, after vs. before
van den Broeke et al. (2019)	Robotic	HFS 20× DT	Forearm	14	64 96	NO NO	136 ± 43^b^ 137 ± 48	NO NO		After vs. before
van den Broeke et al. (2020)	Robotic	HFS 20× DT	Forearm	16	64	YES	136 ± 25	YES	259 ± 40	After vs. before
Scheuren et al. (2020)	Manual	Burn 48°, 6 s	Forearm	20	256	YES	66.4 ± 20.8^c^	YES		Ipsilateral vs. contralateral sites, after vs. before
Gousset et al. (2023)	Robotic	HFS 20× DT	Forearm	20	512	YES	150 ± 30	YES	310 ± 60	After vs. before

^a^ Post‐hoc corrected for trigger delay.

^b^ Baseline control arm.

^c^ Value obtained from the first 15 stimuli applied at baseline in the control condition.

**FIGURE 5:**
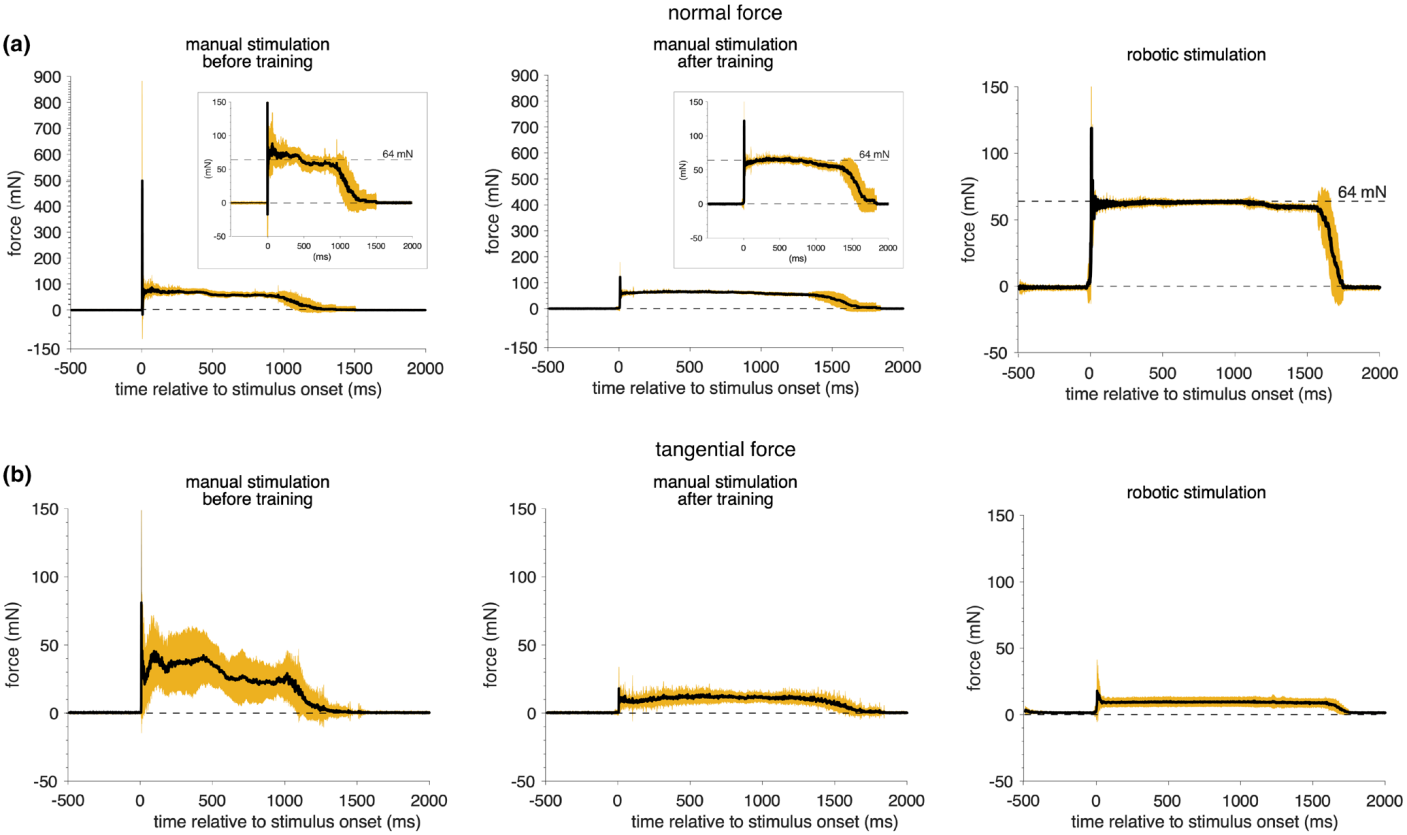
Time course of the force recorded from a force sensor during manually or robotically delivered 64 mN pinprick stimuli on a force sensor ([mean ± SD]). (a) The normal force (along the perpendicular axis of the stimulation) and (b) the tangential force (perpendicular to the axis of stimulation) were recorded during manual or robotic delivery of 30 pinprick stimuli (64 mN) on a 6‐axis strain‐gauge force‐torque transducer. In the manual condition, the experimenter delivered the pinprick stimuli before and after a training period. The training aimed at improving the perpendicular orientation of the pinprick stimulator with respect to the skin with minimal lateral movements (tangential force), and at as constant as possible speed, with a contact held for approximately 1 s. Note after training the reduced overshoot of normal force at the onset of the stimulation. By comparing the inserts in (a), note after training the more constant normal force of 64 mN applied during stimulation and its reduced variability. In (b), note after training that the tangential force is reduced by a factor of 10. After training both lateral and tangential force values induced by manual stimulation are closer to the forces induced by robotic stimulation. Robot‐controlled pinprick stimulation leads to even less variable normal and tangential force and offers the advantage of constant stimulation over time in contrast to the performance of a human experimenter that could suffer from its fatigue throughout an experiment. (Unpublished data Lambert et al., 2017)

To make mechanical pinprick stimulation operator‐independent and to reduce across trial variability of the applied force, van den Broeke et al. (2020) developed a robot‐controlled mechanical pinprick stimulator. Indeed, as can be seen in Figure 5, the applied normal and tangential forces when the pinprick stimuli are applied manually show much more variability across trials compared to pinprick stimuli delivered with the robot‐controlled pinprick stimulator. When robot‐controlled mechanical pinprick stimuli were used to elicit PEPs, van den Broeke et al. (2020) found that the PEP negative peak reached its maximum at around 136 ms and the positive amplitude at around 250 . When applying the pinprick stimuli within the area of increase pinprick sensitivity they found an increase in the amplitude of both peaks (Gousset et al., 2023; van den Broeke et al., 2020). However, one study (van den Broeke et al., 2019) did not observe an increase in the amplitude of PEP peaks after HFS, despite using robot‐controlled mechanical pinprick stimulation. In that study, participants provided a single average rating of all pinprick stimuli at the end of each block. In the studies where an increase in the amplitude of PEPs was observed, the task was different: participants had to rate each single pinprick stimulus individually. It could be that providing a rating only at the end of the block may have rendered the pinprick stimuli less task relevant. One possibility, although speculative, might be that the reduced task relevance prevented the increase in PEPs. However, this should be tested in future studies.

It has been shown that vibrotactile stimuli—predominantly activating LTMs—delivered to the area of SHA elicited an increase in the magnitude of the negative amplitude of the vibrotactile evoked potential reaching its maximum around 130 ms (van den Broeke & Mouraux, 2014a). The positive amplitude elicited by vibrotactile stimuli was not increased and no changes were observed in the perceived intensity of these stimuli. These results suggest that part of the increase in the negative component of PEPs could be modality unspecific whereas the increase in the positive component could be modality specific.

No correlations have been observed between the increase in perceived intensity elicited by pinprick stimuli in the area of SHA and the changes in the amplitude of the negative and positive peaks of PEPs (van den Broeke et al., 2020). Nevertheless, the increase in pinprick perception elicited from the area of SHA tends to follow the same time course as the increase in PEP positivity (van den Broeke, Lambert, et al., 2016). No study has assessed the test–retest reliability, sensitivity and specificity of the increase in PEPs in the context of SHA.

Triccas et al. assessed the test–retest reliability of the negative–positive peak recorded at Cz and elicited by 256 mN pinprick stimulation delivered to the hand dorsum. They found an intra‐class correlation coefficient (ICC and 95% CI) of 0.573 (0.151, 0.897) for the right hand and 0.624 (0.262, 0.832) for the left hand (Triccas et al., 2022). Also, Rosner et al. (2020) assessed the test–retest reliability of the negative–positive peak at Cz after 256 mN pinprick stimulation delivered to the hand dorsum and found an ICC of 0.27.

#### 4.3.1 Pinprick‐evoked EEG responses analysed in the time‐frequency domain

When analysing mechanical pinprick‐evoked EEG responses in the time‐frequency domain, van den Broeke et al. (2017, 2019) and Triccas et al. (2022) found that pinprick stimuli elicited a phase‐locked low‐frequency (<5 Hz) response between 150 and 400 ms after stimulus onset, followed by a reduction of alpha‐band activity (7–10 Hz) between 100 and 600 ms. Triccas et al. (2022) assessed test–retest reliability and found that the low‐frequency response had an almost perfect relative reliability (ICC = 0.98), which was not the case for PEP amplitudes.

van den Broeke et al. (2017) showed that mechanical pinprick stimuli, when applied to the area of increased pinprick sensitivity, elicited an increase in the low‐frequency response but did not change alpha‐band activity. They further showed that the part of the low‐frequency response was phase‐locked to the stimulus and, thus, mostly reflected PEPs. In a later study, van den Broeke et al. (2019) did not find a significant increase in the low‐frequency response when the pinprick stimuli were applied in the area of increased pinprick sensitivity, which is in line with the lack of increase in PEPs as discussed in Section 3.1.3. Future studies should assess the reliability of the increase in low‐frequency response.

Gousset et al. recently investigated if scalp‐recorded gamma‐band activity (30–100 Hz) elicited by strong pinprick stimulation (512 mN) is associated with SHA (Gousset et al., 2023). However, they only observed pinprick‐evoked gamma‐band activity in a subgroup of participants and this activity was not significantly increased when pinprick stimuli were applied to the area of SHA. This conclusion is supported by a re‐analysis of the data using different parameters (Jaltare et al., 2023).

Also in a previous study, van den Broeke et al. (2017) assessed if manually applied 64 mN pinprick stimulation to the area of SHA increased scalp‐recorded gamma‐band activity. However, the authors did not observe any increase in cortical‐related gamma‐band activity after HFS.

### 4.4 Brainstem autonomic activity assessed by pupil dilation response

Pupil size has been shown to reflect in primates the activity of neurons of the locus coeruleus (Joshi et al., 2016) which is thought to play an important role in the regulation of arousal (Berridge, 2008) and which activity is increased in response to a nociceptive stimulus (Samuels & Szabadi, 2008).

van den Broeke et al. (2019) were the first to record the pupil dilation response (PDR) elicited by robot‐controlled mechanical pinprick stimulation before and after the experimental induction of SHA using HFS. They found that pinprick stimulation (64 and 96 mN) applied to non‐sensitized skin of the ventral forearm elicited a dilation of the pupil that reached its maximum between 1 and 2 s after stimulus onset. They further showed that when the same pinprick stimuli were delivered to the area of SHA the pupil dilated even more. No increase in pupil dilation response was observed at the control site (contralateral arm) after the induction of SHA. However, the PDR elicited by the 64 mN delivered to the area of SHA was on average larger than the one elicited by the 96 mN, while the increase in perceived intensity was on average similar between both pinprick stimulation intensities, suggesting that there was no correlation between the two variables. This finding should be interpreted with caution as the sample of participants was small.

Of note, the increase in PDR associated with SHA was present without any accompanying increase in PEPs, suggesting that the PDR is more sensitive (or less dependent on task relevance as discussed for PEPs in Section 3.1.3) to detect changes in central nervous system processing of mechanical nociceptive input than PEPs. No studies have assessed the test–retest reliability of the increase in PDR associated with SHA and no data on the specificity and sensitivity are available.

### 4.5 Brainstem autonomic activity assessed by sympathetic skin response

The sympathetic skin response (SSR) is measured as a change in electric potential, in relation to the modification of skin conductance due to sweat gland's activity, in response to a stimulus (Vetrugno et al., 2003). SSR has been used as a means to indirectly assess the changes in the autonomic nervous system activity, and in particular of the sympathetic tone, in the context of sensitization. Scheuren et al. (2020) recorded the SSR elicited by pinprick stimulation (256 mN) before and after the induction of SHA using repetitive heat stimulation. Together with an increase in pinprick ratings, they found increased amplitudes of the SSR in response to pinprick stimulation applied in the area of SHA compared to both baseline and control conditions.

In a follow‐up study, the same group did not replicate this increase in SSR elicited by pinprick stimulation in the area of SHA but found a lack of habituation (compared to the control condition) only (Scheuren et al., 2023). However, in the second study the pain intensity of all pinprick and heat stimuli was individually adapted to NRS‐4, therefore the two studies are not directly comparable. Interestingly, also heat stimuli applied to the area of SHA elicited a lack of habituation of the SSR amplitude.

No data is available about the test–retest reliability of the increase in SSR nor about sensitivity and specificity.

## 5 DISCUSSION

### 5.1 Experimentally induced central sensitization involves predominantly a facilitation of mechanical nociceptive pathways

#### 5.1.1 Heat sensitivity

The presence of heat hyperalgesia seems to depend on the method used to induce SHA (intradermal capsaicin and HFS) and the stimulus to assess it (Table 1). There is no obvious explanation for this observation. The fact that heat hyperalgesia manifests with the use of experimental models that induce greater pain (Quesada et al., 2021) leads us to think that the intensity of the conditioning stimulus (peripheral nociceptive input) and the characteristics of the type of afferent might play a role.

#### 5.1.2 Do tactile afferents play a role in SHA?

The quality and perceived intensity elicited by static indenting non‐nociceptive tactile stimuli are not changed in the area of SHA. This suggests that A‐fibre low threshold mechanoreceptors may have a negligible contribution to SHA. This conclusion does not preclude the involvement of LTMs in dynamic mechanical allodynia (Baron, 2000; Cervero et al., 1994; Pfau et al., 2011; Simone & Ochoa, 1991).

#### 5.1.3 Can we detect SHA using electrical stimuli?

Our findings suggest that changes in perceived intensity to electrical stimuli in the SHA area can vary with stimulus characteristics. In the case of suprathreshold electrical stimuli (above pain threshold), the change involves a difference compared to the control site rather than an increase compared to baseline. According to the dual process theory proposed by Groves and Thompson (1970), habituation and sensitization processes interact, leading to the net outcome of behaviour. A difference between arms but not between before and after, might suggest either reduced habituation or weak sensitization.

Low‐intensity intraepidermal stimulation, which has been shown to selectively activate A‐fibre nociceptors, does not detect secondary mechanical hyperalgesia.

High‐intensity electrical stimuli (which are non‐selective for activating types of primary afferents) can increase perception, possibly due to the synchronous activation of many fibres. This synchronous recruitment might be perceived differently than natural mechanoreceptor activation, leading to distinct perceptual experiences (Graczyk et al., 2016).

Currently, it is impossible to conclude which nerve fibres mediate SHA from transcutaneous electrical stimulation results.

### 5.2 Physiological measures, what do they reflect?

#### 5.2.1 RIII reflex and the N13 SSEP

During RIII recording, distal electrical stimulation activates small‐myelinated fibres, transmitting afferent input to the spinal cord dorsal horn. Interneurons, with a primary role of WDR, are involved, as shown in animal studies (Li & Chen, 2004). The output connects to motor neurons (Lundberg, 1979), and the RIII is subject to dynamic supraspinal modulation (Sandrini et al., 2005). Given the complexity of the reflex generation, it is difficult to establish how and which of these neurons are facilitated during the induction of SHA. While the threshold of the reflex is systematically decreased after different SHA induction methods, the reflex AUC appears to be modulated only if a continuous afferent input, whether this is due to the presence of continuous pain or by mimicking the primary afferents firing rate through a long‐lasting LFS, is provided to the spinal cord (Table 3), suggesting that the two outcomes are not equally affected in sensitized contexts.

The N13 does not reflect the activity of spinal nociceptive specific neurons (Restuccia & Mauguière, 1991). However, evidence suggest that this response might reflect the activity of WDR neurons. It was shown to be increased in amplitude by experimental methods inducing SHA through a prolonged peripheral nociceptive input, making it a suitable readout of spinal excitability changes (Di Lionardo et al., 2021; Leone et al., 2023). However, the low signal‐to‐noise ratio, the small amplitude of the response, the dependence of its modulation on specific experimental methods and the limited data available on its test–retest reliability imply that this neurophysiological tool is still in its infancy and requires further studies. Nevertheless, the N13 recorded at the group level might be useful to assess drug effects on dorsal horn excitability in pharmacological trials. Future studies should replicate the modulation of this response by selected pain methods used to induce CS.

Based on this information, we can conclude that the threshold of the RIII reflex is a reliable readout of spinal changes occurring in the context of SHA induction. However, modulation of the reflex AUC only occurs when there is ongoing nociceptive afferent input to the spinal cord.

#### 5.2.2 Pinprick‐evoked potentials

Studies have shown that mechanical pinprick stimuli delivered to the area of SHA elicit an increase in the amplitude of both the negative and positive peaks of PEPs. The available data suggest that the increase in the positive peak is stimulus specific and site specific, though it seems to be task dependent. Data suggest that pinprick‐evoked gamma‐band activity is not useful for assessing altered processing of mechanical nociceptive input in the context of SHA. One study has found that the pinprick‐evoked low‐frequency EEG response (<5 Hz) has a high reliability and therefore future studies should investigate the reliability of the increase of this low‐frequency response in the context of SHA.

As sensitivity and specificity are unknown, the usefulness of these EEG features for detecting CS remains to be clarified.

#### 5.2.3 Pupil dilation response and sympathetic skin response

The PDR elicited by mechano‐nociceptive stimuli may reflect changes in locus coeruleus, a brainstem structure thought to be also involved in arousal (Joshi et al., 2016). Mechanical stimuli delivered to the area of SHA elicit a larger PDR as compared to pinprick stimuli delivered before sensitization or compared to the control site. Interestingly, whereas the increase in PEPs seems task‐dependent, the PDR is less (van den Broeke et al., 2019).

The SSR is increased when elicited both by mechanical pinprick and heat stimulation delivered to the area of SHA (Scheuren et al., 2020, 2023), indicating that SSR modulation is not modality specific and more related to a general increase in the state of arousal.

### 5.3 Lack of association between changes in perception and physiological measures

Correlations between changes in perception and neurophysiological responses are not systematically observed, indicating that there is no one‐to‐one relationship between them. The fact that various experimental pain models seem to elicit different effects with variable time courses supports the idea of multiple pathways involved (Di Lionardo et al., 2021; Gousset et al., 2020). In addition to the long‐lasting sensitization of mechanical nociceptive pathways, pain models also appear to induce short‐term changes that are not specific to a particular sensory modality. These short‐term changes could result from higher‐level cognitive processes (van den Broeke et al., 2014) or reflect changes in spinal excitability unrelated to changes in mechanical pinprick processing (Leone et al., 2023).

### 5.4 Different methods to induce CS might differently affect spinal circuitries

All revised experimental pain methods trigger an increased pinprick sensitivity in the surrounding skin, but they impact behavioural and spinal responses differently. This indicates that the different experimental pain stimuli may affect spinal circuits differently. Change in heat sensitivity, for example, is more common with intense pain during the experimental model (Quesada et al., 2021). The amplitudes of spinal‐evoked responses change only with prolonged spinal afferent input. Since hyperexcitability in spinal neurons has been shown mainly with specific methods such as intradermal capsaicin and HFS (Patel et al., 2024; Randić et al., 1993; Simone & Ochoa, 1991), it questions whether other models can cause similar dorsal horn changes. The different experimental pain methods might explain some inconsistent findings. Future research should use methods with better‐defined spinal mechanisms to induce SHA, such as capsaicin and electrical stimulation.

### 5.5 Methodological considerations and recommendations

The use of numerical rating scales (NRS) is known to generate non‐ratio scales as opposed to visual analogue scales (VAS) (Myles & Urquhart, 2005; Price et al., 1983, 1994). Therefore, to obtain continuous data with similar mathematical properties that could be analysed with standard statistical methods and be compared across studies, the use of VAS should be preferred. To standardize the rating scales and their use, importance should be given to the definition of the anchors. The use of an intensity VAS in addition to a quality assessment using descriptors of the sensation should be preferred. During assessment at multiple time points, keeping visible the previous VAS used by the participant could allow him/her to have a reference for subsequent ratings without relying on the memory of the perception elicited by previous stimuli. A precise description of the instruction should systematically be reported to standardize experimental setups and allow fair comparisons between studies. Some other factors might play an important role such as the testing order between different modalities, and the body sites explored (Leone et al., 2021).

Studies in this review required SHA to be evidenced by pinprick stimuli. SHA assessment involves increased perceived pinprick intensity and an area. Research shows that while pinprick sensitivity generally increases across methods (except UVB models) the area of hyperalgesia can vary by stimulation quality, intensity and application site (Quesada et al., 2021). These findings highlight how increased pinprick sensitivity around the pain application site is an essential indicator of pain model success. Accurately mapping the hyperalgesia area before collecting pinprick ratings is crucial to minimize biases.

## 6 CONCLUSION

The most reliable behavioural readout for SHA is pinprick hyperalgesia. This should ideally be assessed using visual rating scales, after outlining the hyperalgesic area. Currently, the best neurophysiological readout for evaluating spinal hyperexcitability induced by sensitizing methods is the RIII reflex threshold. Animal studies with direct recordings from the dorsal horn are necessary to clarify the excitability mechanisms induced by different experimental models. Until such data is available, it is preferable to use methods whose mechanisms are at least partially understood and reproducible, that is, methods such as topical application or injection of capsaicin and electrical stimulation of the skin.

Test–retest reliability, sensitivity and specificity data of all the revised neurophysiological outcomes are scarce, therefore none of these methods is currently indicated for CS identification at the individual level. The clinical use is further limited by the fact that a comparison between the healthy side and the affected side in patients is not possible given the fact that central sensitization thought to be linked to persistent pain might be widespread (Arendt‐Nielsen et al., 2018). It is limited, at present, to the comparison between populations. It is worth mentioning that Quantitative Sensory Testing according to DFNS standards including clustering (Vollert et al., 2017) has the characteristics to provide reliable information at the individual level. This battery of tests was beyond the scope of this review as it does not constitute an objective neurophysiological tool.

## AUTHOR CONTRIBUTIONS

The authors contributed equally to the review by revising papers, drafting the manuscript and discussing the results.
